# Effects of pre-exercise H_2_ inhalation on physical fatigue and related prefrontal cortex activation during and after high-intensity exercise

**DOI:** 10.3389/fphys.2022.988028

**Published:** 2022-09-02

**Authors:** Yinglu Hong, Gengxin Dong, Qian Li, Vienna Wang, Meng Liu, Guole Jiang, Dapeng Bao, Junhong Zhou

**Affiliations:** ^1^ School of Sport Medicine and Physical Therapy, Beijing Sport University, Beijing, China; ^2^ Sports Coaching College, Beijing Sport University, Beijing, China; ^3^ California State University, Long Beach, CA, United States; ^4^ National University of Defense Technology, Changsha, Hunan, China; ^5^ China Institute of Sport and Health Science, Beijing Sport University, Beijing, China; ^6^ Hebrew Senior Life Hinda and Arthur Marcus Institute for Aging Research, Harvard Medical School, Boston, MA, United States

**Keywords:** physical fatigue, prefrontal cortex, H_2_ gas, fNIRS, anti-oxidation

## Abstract

**Objective:** In this study, we examined the effects of pre-exercise H_2_ gas inhalation on physical fatigue (PF) and prefrontal cortex (PFC) activation during and after high-intensity cycling exercise.

**Methods:** Twenty-four young men completed four study visits. On the first two visits, the maximum workload (W_max_) of cycling exercise of each participant was determined. On each of the other two visits, participants inhaled 20 min of either H_2_ gas or placebo gas after a baseline test of maximal voluntary isometric contraction (MVIC) of thigh. Then participants performed cycling exercise under their maximum workload. Ratings of perceived exertion (RPE), heart rate (HR) and the PFC activation by using functional near-infrared spectroscopy (fNIRS) was measured throughout cycling exercise. The MVIC was measured again after the cycling.

**Results:** It was observed that compared to control, after inhaling H_2_ gas, participants had significantly lower RPE at each workload phase (*p* < 0.032) and lower HR at 50% W_max_, 75% W_max_, and 100% W_max_ during cycling exercise (*p* < 0.037); the PFC activation was also significantly increased at 75 and 100% W_max_ (*p* < 0.011). Moreover, the H_2_-induced changes in PF were significantly associated with that in PFC activation, that is, those who had higher PFC activation had lower RPE at 75% W_max_ (*p* = 0.010) and lower HR at 100% W_ma*x*_ (*p* = 0.016), respectively.

**Conclusion:** This study demonstrated that pre-exercise inhalation of H_2_ gas can alleviate PF, potentially by maintaining high PFC activation during high-intensity exercise in healthy young adults.

## Introduction

Physical fatigue (PF) is common during exercise, especially during the vigorous exercise with prolonged periods (Cordeiro et al., 2017). The PF oftentimes results in diminished sport performance (Birfer et al., 2019) and increased risk of athletic injury (Huygaerts et al., 2020). Therefore, strategies to alleviate the burden of PF are critical to improve sport performance and reduce risk of injury.

The development of PF is not only associated with peripheral factors in human neurophysiological procedures (e.g., failure of muscle contractility and excitatory contraction (E-C) coupling (Allen et al., 2008)) (Gandevia, 2001; Rasmussen et al., 2007), but also depends upon the altered regulation of the components in central nervous system (Enoka, 1995; Siemionow et al., 2000). For example, the prefrontal cortex (PFC) of the brain is thought to be the place where the information pertaining to fatigue is processed and the command to terminate the continuation of exercise is initiated (St Clair Gibson et al., 2013; Robertson and Marino, 2016; Hyland-Monks et al., 2018). Studies have observed that the activation of PFC, which can be reliably measured by the oxygenation using functional near-infrared spectroscopy (fNIRS), is linked to the degree of fatigue, that is, greater fatigue is associated with lower PFC activation (De Wachter et al., 2021). The capacity to maintaining the activation of PFC is thus critical to alleviate fatigue and maintain the athletic performance. For example, it was observed that the elite Kenyan runners with average time of 62.2 min to complete 21-km race can maintain PFC activation during a self-paced 5-km test, which may contribute to their success of long-distance running competition (Santos-Concejero et al., 2015). Therefore, strategies that can modulate PFC activation may be of great promise to help fatigue attenuation.

Recently, hydrogen molecules (H_2_) has gained much attention because of its biological effects (Kawamura et al., 2020) that can benefit brain function (Mizuno et al., 2017). The brain is susceptible to damage from reactive oxygen species (ROS) (Cobley et al., 2018) which often increase dramatically along with the increase of the demand for energy and metabolism, especially during high-intensity exercise (Fisher-Wellman and Bloomer, 2009; Kawamura and Muraoka, 2018). The H_2_ acts as a powerful antioxidant that can cross the blood-brain barrier (Hayashida et al., 2012) and then protect neuronal cells by selectively eliminating harmful ROS (Tomofuji et al., 2014; McCarty, 2015). In a double-blinded, placebo-controlled study, 4 weeks administration of hydrogen-rich water (HRW) helped reduce and prevent accumulated oxidative stress in the brain, thereby improving mood, anxiety, and autonomic function in adult volunteers (Mizuno et al., 2017). Recently, several studies have arisen to explore the potential anti-fatigue effects of H_2_ (Table 1) in healthy cohorts who performed either acute or chronic exercise, and shown the promise of intaking H_2_ either before or after the exercise may help alleviate fatigue (Aoki et al., 2012; Da Ponte et al., 2018; Botek et al., 2019; LeBaron et al., 2019; Mikami et al., 2019; Dobashi et al., 2020; Hori et al., 2020; Shibayama et al., 2020; Timon Andrada et al., 2020; Dong et al., 2022). For example, intaking HRW before exercise has been shown to improve exercise-induced decline of muscle function (Aoki et al., 2012), and thus alleviate fatigue (Pantovic et al., 2016); and the inhalation of hydrogen-rich gas mixture after exercise can help attenuated the reduction in athletic performance (e.g., the height of countermovement jump) (Shibayama et al., 2020). However, the effects of inhalation of *H*_*2*_ *gas before* high-intensity endurance exercise/fatigue on PF have not been well characterized, and it is still not clear if such benefits of H_2_ are achieved by modulating the cortical characteristics of the brain.

**TABLE 1:** Summary of anti-fatigue effects of H_2_ in human clinical trials.

References	Participants	Type of H_2_	Administration of H_2_	Changes in biomarkers
Aoki et al. (2012)	Male soccer players (n = 10)	HRW	Pre-exercise; Acute supplementation	Blood lactate↓, Peak torque↑
Da Ponte et al. (2018)	Trained male cyclists (n = 8)	HRW	Pre-exercise; 2-weeks	PPO↑, △PPO↑
Mikami et al. (2019)	Healthy non-trained participants (n = 99)	H_2_ water	Pre-exercise; Acute supplementation	Psychometric fatigue↓, Endurance↑, Fatigue judged by Borg’s scale↓
LeBaron et al. (2019)	Healthy subjects (n = 19)	HRW	Pre-exercise; Acute supplementation	average exercising RR and HR↓
Botek et al. (2019)	Healthy males (n = 12)	HRW	Pre-exercise; Acute supplementation	Blood lactate↓, Ventilatory equivalent for oxygen and RPE↓
Dobashi et al. (2020)	Healthy and physically active males (n = 8)	HRW	Pre- and post-exercise; Acute supplementation	Serum BAP/d-ROM↑
Hori et al. (2020)	Healthy participant (n = 9)	HRW	Pre-exercise; 2-weeks	Peak oxygen uptake↑, Peak load↗
Timon Andrada et al. (2020)	Trained and untrained subjects (n = 37)	HRW	Pre-exercise; 7-days	Peak power↑, Mean power↑, Fatigue index↓
Shibayama et al. (2020)	Active male volunteers (n = 8)	HG	Post-exercise; Acute supplementation	Urinary 8-OHdG excretion rate↓, Countermovement, jump height↑
Dong et al. (2022)	Dragon boat athletes (n = 18)	HRW	Pre-exercise; 7-days	MP↑, AP↑, MHR↓, HRPC after 2 min of Recovery↓

Abbreviations: H_2_ = molecular hydrogen; HRW, hydrogen-rich water; PPO, peak power output; RR, respiratory rate; HR, heart rate; RPE, ratings of perceived exertion; BAP, biological antioxidant potential; d-ROM, diacron-reactive oxygen metabolites; HG, hydrogen-rich gas mixture; 8-OHdG = 8-hydroxydeoxyguanosine; MP, maximum power; AP, average power; HRPC, heart rate percent change; ↓ = significant decrease; ↑ = significant increase; ↗ = slight increase.

We here thus contend that pre-exercise inhalation of H_2_ gas can help PF by maintaining high level of PFC activation during high-intensity cycling exercise. To examine this, we here completed a randomized and double-blinded within-subject study in a group of healthy young adults. We hypothesized that as compared to control, pre-exercise inhalation of H_2_ gas would induce lower level of PF and greater activation of PFC; and such H_2_-induced improvement in PF would be associated with that in PFC activation.

## Materials and methods

### Participants

Twenty-four young men were recruited from Beijing Sport University. Inclusion criteria were age 18–35 years, the ability to complete the incremental exercise test and the cycling time to exhaustion prior to the formal trial. Exclusion criteria were self-reported acute illness, injury, or unstable medical condition or hospitalization within the past 3 months; any report of diseases of the musculoskeletal system, pain, or orthopedic problems likely to affect exercise; use of antipsychotics, anti-seizure, or other neuroleptic medication. The baseline characteristics of the participants are shown in Table 2. Before the study visits, participants were instructed to refrain from exercising that might cause fatigue for 48 h and alcohol and caffeine intake for 24 h. All participants were informed of the relevant benefits and possible risks involved in participating in this study and provided signed informed consent in order to participate in this study. The consent form included information contained in the Helsinki Declaration as well as the purpose of the study and details about the study’s protocols. This study was reviewed and approved by the Institutional Review Board of Beijing Sport University (number: 2021163H).

**TABLE 2:** Participants’ physical characteristics (n = 24) (mean ± SD).

Variable	Value
Age (year)	21.33 ± 2.68
Height (cm)	177.38 ± 4.53
Body mass (kg)	70.71 ± 7.26
Body mass index (kg/m^2^)	22.45 ± 1.93
Peak power output (w)	209.17 ± 30.35
Maximal riding time (min)	20.49 ± 8.27

### Experimental design

In this within-subject and double-blinded study, each participant completed four study visits. During the first visit, an incremental exercise test (2 min at 50 W + 20 W increments every 2 min) was performed until exhaustion (operationally defined as a pedal frequency of less than 60 revolutions/min (RPM) for more than 5 s with strong verbal encouragement) on an electromagnetically braked cycle ergometer (Excalibur Sport, Lode, Groningen, Netherlands) to measure peak power output.

**FIGURE 1:**
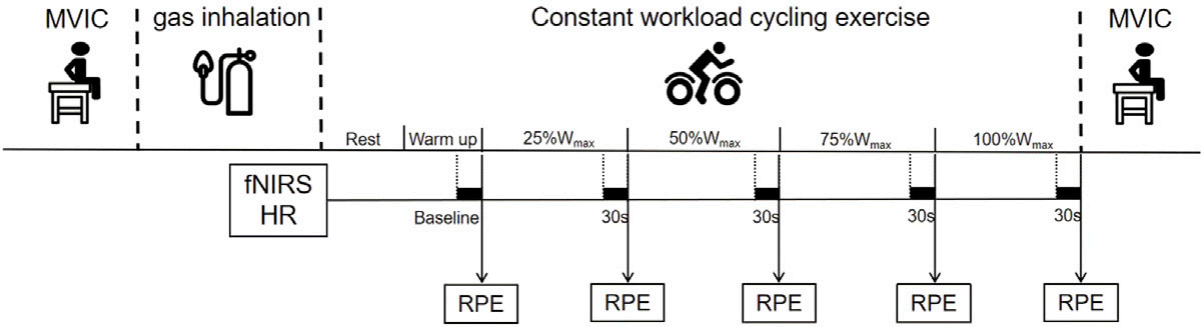
Assessment protocol on one visit (Visit 3 or 4).

After an interval of at least 48 h, on the second visit, participants performed a time-to-exhaustion cycling test to measure the maximum riding time (MRT). The test consisted of a 3 min warm-up at 40% of peak power output followed by a rectangular workload corresponding to 80% of peak power output that was achieved in the first visit. The test was completed when the pedal frequency was less than 60 RPM for more than 5 s despite standardized verbal encouragement. On the next two visits, participants completed the exercise and functional assessments after the H_2_ intervention. These two visits were separated by 1 week (i.e., wash-out period) to avoid potential after effects of the interventions from the prior visit (Javorac et al., 2019). Specifically, on each of the two visits, participants inhaled H_2_ gas (H_2_ group) or placebo gas (PLAC group) for 20 min when seating quietly in a chair, respectively, in a randomized order. The duration of H_2_ inhalation was 20 min, as determined according to the observations from previous studies (Korovljev et al., 2018; Javorac et al., 2019). Before the inhalation, participants completed the maximal voluntary isometric contraction (MVIC) test. Immediately after gas inhalation, participants sat quietly on the cycle ergometer for 2 min to complete the baseline assessment of resting-state fNIRS. Then after warming up at a load of 40% peak power output for 3 min, participants were instructed to ride at 80% peak power output for the duration of their MRT. The Borg CR10 Scale was used to quantify the whole-body fatigue during cycling. Heart rate (HR) and oxygenation of the PFC were recorded throughout the exercise (the averaged values of the last 30 s of the 0% (baseline), 25%, 50%, 75% and 100% of the maximum workload (W_max_) phases were intercepted for statistical analysis, respectively). After the ride, participants repeated MVIC test. The overview of the assessment protocol on one visit (Visit 3 or 4) is shown in Figure 1.

### H_2_ gas

H_2_ gas was supplied through a hydrogen oxygen supply unit (Zhiheng Hydrogen Health Technology Co., Ltd., Fuzhou, China) connected to a nasal inhaler. The ratio of oxygen to hydrogen in the H_2_ gas is 2:1, and the maximum concentrations are 21.57% and 4.08%, respectively (Javorac et al., 2019).The flow rate of H_2_ gas is 1800 ml/min. The placebo gas was supplied by the same hydrogen oxygen supply unit that did not initiate the hydrogen production program. Participants reported no different feelings on the gas between the two inhalations.

### Ratings of perceived exertion

Ratings of Perceived Exertion (RPE) was defined as the intensity of subjective effort, exertion, discomfort, or fatigue felt during exercise (Borg, 1982), as measured by the Borg CR10. The RPE represented feedback from cardiovascular, respiratory and musculoskeletal systems and it provides a whole-body fatigue assessment, while a single physiological parameter usually provides very limited information (Crewe et al., 2008; Venhorst et al., 2018). We here recorded the RPE of participants at the end of each workload phase (i.e., at baseline, 25%W_max_, 50% W_max_, 75% W_max_ and 100% W_max_) and used it as the primary outcome of PF.

### Heart rate monitoring

We used the Firstbeat heart rate belt (Firstbeat Analytics, Jyvaskyla, Finland) to monitor the HR changes of the participants during the cycling exercise. The heart rate belt was worn on the participant’s chest, and the receiver was positioned to the left of the midline. The elastic band was adjusted so that the position of the receiver did not change during the ride. During exercise, especially the prolonged high-intensity exercise, cardiovascular restriction is an important factor leading to fatigue (Nybo et al., 2014). Since HR is linearly related to exercise intensity (Portier et al., 2001; James et al., 2002), lower HR at the same exercise intensity represent the ability of the cardiovascular system to adapt to fatigue (LeBlanc et al., 1977). Therefore, HR was used as one of the secondary outcomes of PF in this study.

### The maximum voluntary isometric contraction test

The MVIC was measured by a LINK isometric dynamometer (KFORCE, KINVENT BIOMECANIQUE, France). The participants were seated at the edge of the treatment bed with straps securing the trunk and thighs to avoid generating compensatory force. Then participants performed MVIC tests for three times at a knee joint angle of 60° with a 5 s interval. Each test lasted 5 s, and we took the average of these 5 s as the result of each test and the average of three tests was identified as the MVIC. Muscle fatigue is an exercise-induced reduction in maximal voluntary muscle force (Gandevia, 2001). We here quantified muscle fatigue using the change in MVIC before and after exercise (ΔMVIC) and used it as one of the secondary outcomes of PF.

### Measurement of fNIRS

For PFC activation, the hemodynamic response of prefrontal brain areas was recorded using a multichannel continuous-wave fNIRS device (Oxymon, Artinis, Netherlands) consisting of 10 light sources and eight detectors mounted on a head cap. The head cap placement was centered around Cz (10/20 international system for electrode placement), the mid-point between the nasion to inion and left to right preauricular distances. Light sources and detectors were placed in the F4-FP1-F3 of the PFC area, to ensure consistency between participants and sessions. Data is collected at a frequency of 10 Hz.

Prior to the ride, participants were asked to sit quietly for 2 min with eyes open, breathing normally, relaxed and not controlling their mental activity in any particular way. The data recorded in the last 30 s were used as the baseline of resting-state fNIRS. Similarly, the average values of the last 30 s of each workload phase as described above (Figure 1) were intercepted respectively. Relative concentration changes (Δμmol) of oxyhemoglobin (ΔO_2_Hb), deoxyhemoglobin (ΔHHb), and total hemoglobin (ΔTHb = ΔO_2_Hb + ΔHHb) from baseline of resting-state to each workload were obtained to infer neural activation. Among them, △O_2_Hb was presumed to be the metabolic adjustment in response to neural firing (Obrig and Villringer, 2003) and is the most sensitive indicator of regional brain oxygenation changes and neural activation (Hoshi et al., 2001; Pan et al., 2019), which has been widely used to assess the changes in cortical neural activation during exercise (Jung et al., 2015; Radel et al., 2017). Therefore, we here used △O_2_Hb as the primary outcome of the PFC activation.

### Data processing of fNIRS

The raw fNIRS data were preprocessed using the NIRS_KIT toolkit, an open-source package in MATLAB. To estimate the quality of the data channel, the signal-to-noise ratio of them was measured. First, the relative coefficient of variation (CV in %) was calculated for the raw data before filtering, which is a widely-used method for measuring multichannel NIRS (Schmitz et al., 2005; Schneider et al., 2011). The CV of each channel was calculated over the entire experimental duration, and CVs above 15% were rejected (Piper et al., 2014). Then the data was pre-processed using a third order low-pass filter of 0.2 Hz to remove non-related physiological components (e.g., heartbeats: 0.5–2.0 Hz, and respiration: 0.2–0.4 Hz) (Scholkmann et al., 2014), and then removed slow drift.

### Statistical analysis

Statistical analyses were performed using SPSS 26.0 (IBM, Chicago, IL, United States). The significance level was set at *p* ≤ 0.05. Descriptive statistics (i.e., mean, standard deviation (SD)) were used to summarize the demographic characteristics of the participants and study outcomes.

To examine the effects of H_2_ gas on PF and PFC activation, two-way repeated-measures analyses of variance (ANOVAs) models were used. The dependent variable of each model was the primary outcomes (i.e., RPE) and the model factors were group (i.e., PLAC and H_2_ group) and workload (i.e., 0%, 25%, 50%, 75% and 100%W_max_) and their interactions. Similar analyses were used to examine such effects on the secondary outcome (i.e., HR). A Fisher’s LSD Post-hoc analyses were performed if there were significant interactions. As the MVIC was measured before the inhalation and after the exercise, we used one-way ANCOVA to compare the effects of H_2_ gas on it. The model factor was group and the pre-exercise MVIC was included as the covariate in the model.

To examine the associations between the H_2_-induced significant changes in PF outcomes (e.g., RPE and HR) and that in PFC activation (e.g., △O_2_Hb), we first calculated the percent changes of each outcome that was significantly different between groups (see Results below) from PLAC to H_2_ gas group for each participant. We then used the linear regression model to examine such associations.

## Results

All 24 participants completed all experiments. However, the signal quality of the fNIRS channel was poor in 3 participants at the time of the experiment, so the fNIRS data of these 3 participants were not included in the statistical analysis. In addition, a total of eight HR data sets (five in PLAC group and two in H_2_ group) had to be excluded from the statistical analysis due to incomplete HR recorded by the heart rate band. No significant differences in RPE, HR and fNIRS outcomes at baseline were observed between PLAC and H_2_ gas inhalation (*p* > 0.131), but at baseline the MVIC was inconsistent in H_2_ and PLAC group (47.61 ± 2.87 vs. 53.44 ± 3.10; *p* = 0.006).

### The effects of H_2_ gas on physical fatigue

Primarily, two-way mixed design ANOVA model showed a significant interaction of group and workload (F = 3.328, *p* = 0.026) on RPE. The post-hoc analysis showed that: 1) compared to baseline, RPE in both groups were significantly increased when workloads increased, suggesting the success of fatigue construction (*p* < 0.001); 2) and compared to PLAC group, RPE was significantly smaller in H_2_ group at each workload phase (*p* < 0.032) (Table 3).

**TABLE 3:** RPE, HR and PFC activation during the cycling exercise in the PLAC and H_2_ groups (mean ± SD).

	PLAC group					H_2_ group				
Variable	Baseline	25% W_max_	50% W_max_	75% W_max_	100%W_max_	Baseline	25% W_max_	50% W_max_	75% W_max_	100%W_max_
RPE	1.91 ± 0.87	4.83 ± 1.88	7.04 ± 2.07	9.25 ± 1.80	10.68 ± 0.90	1.88 ± 0.36	3.95 ± 1.37^*^	6.25 ± 1.70^*^	8.04 ± 1.68^**^	9.54 ± 1.59^**^
HR	114.68 ± 10.64	157.84 ± 11.92	169.47 ± 11.60	176.16 ± 8.76	179.53 ± 9.03	111.74 ± 18.64	149.37 ± 18.92	159.79 ± 15.69^*^	166.58 ± 11.33^**^	155.68 ± 18.88^**^
PFC activation										
△O_2_Hb	-2.27 ± 7.27	-0.97 ± 5.74	-0.59 ± 6.26	-2.90 ± 8.57	-4.12 ± 8.50	1.14 ± 7.07	0.10 ± 2.41	0.92 ± 3.99	2.39 ± 6.38^*^	3.24 ± 7.22^**^
△HHb	0.35 ± 1.54	0.77 ± 2.19	1.43 ± 3.82	1.50 ± 4.39	0.77 ± 5.61	0.23 ± 2.91	-0.17 ± 3.10	-0.22 ± 5.14	1.89 ± 3.93	3.13 ± 4.57
△THb	-0.35 ± 4.74	0.93 ± 6.49	2.68 ± 10.37	1.80 ± 9.69	-0.72 ± 11.36	1.80 ± 10.02	-0.03 ± 6.34	1.58 ± 6.64	1.88 ± 15.77	1.96 ± 6.80

Note: * Significant differences compared with the PLAC, group under the same workload, * = *p* < 0.05, ** = *p* < 0.01. Abbreviations: RPE, ratings of perceived exertion; HR, heart rate; O_2_Hb = oxyhemoglobin; HHb: = deoxyhemoglobin; THb, total hemoglobin; W_max_ = maximum workload.

Additionally*,* two-way mixed design ANOVA model showed an interaction of group and workload on HR (F = 5.616, *p* = 0.007). The post-hoc analysis showed that the HR of the H_2_ group was significantly smaller than that of the PLAC group at 50%, 75% and 100% of the maximum workload phase (*p* < 0.037) (Table 3). The One-way ANCOVA model showed that after adjusting the pre-test MVIC, there was only a marginally significant difference between the post-test MVIC in H_2_ gas and PLAC group (34.38 ± 1.76 vs. 34.03 ± 1.84; F = 3.341, *p* = 0.074).

### The effects of H_2_ gas on PFC activation

**FIGURE 2:**
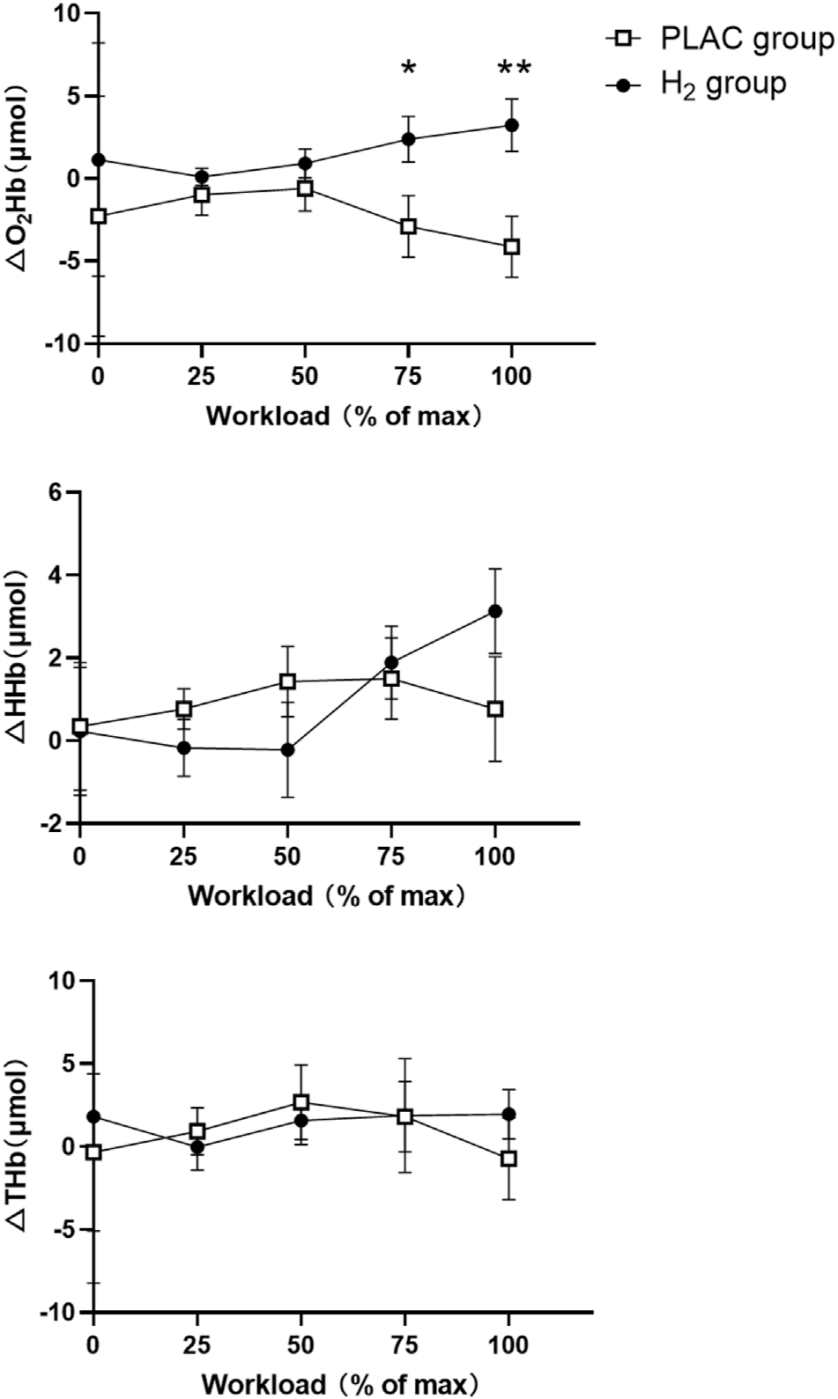
PFC activation during the cycling exercise in the PLAC and H_2_ groups. Note: * Significant differences compared with the PLAC group under the same workload, * = *p* < 0.05, ** = *p* < 0.01. Abbreviations: O_2_Hb = oxyhemoglobin; HHb = deoxyhemoglobin; THb = total hemoglobin.

Two-way mixed design ANOVA model showed a significant interaction of group and workload on △O_2_Hb (F = 3.357, *p* = 0.030). The post-hoc analysis showed that the △O_2_Hb in H_2_ group was significantly higher than that of the PLAC group at 75%W_max_ and 100%W_max_ (*p* < 0.011). Similarly, a significant interaction of △HHb was also observed (F = 4.076, *p* = 0.016); however, only marginally significant difference of it between H_2_ and PLAC groups at 100%W_max_ (*p* = 0.058) was observed and not any other significance was presented (Table 3; Figure 2).

### The associations between physical fatigue and PFC activation

**FIGURE 3:**
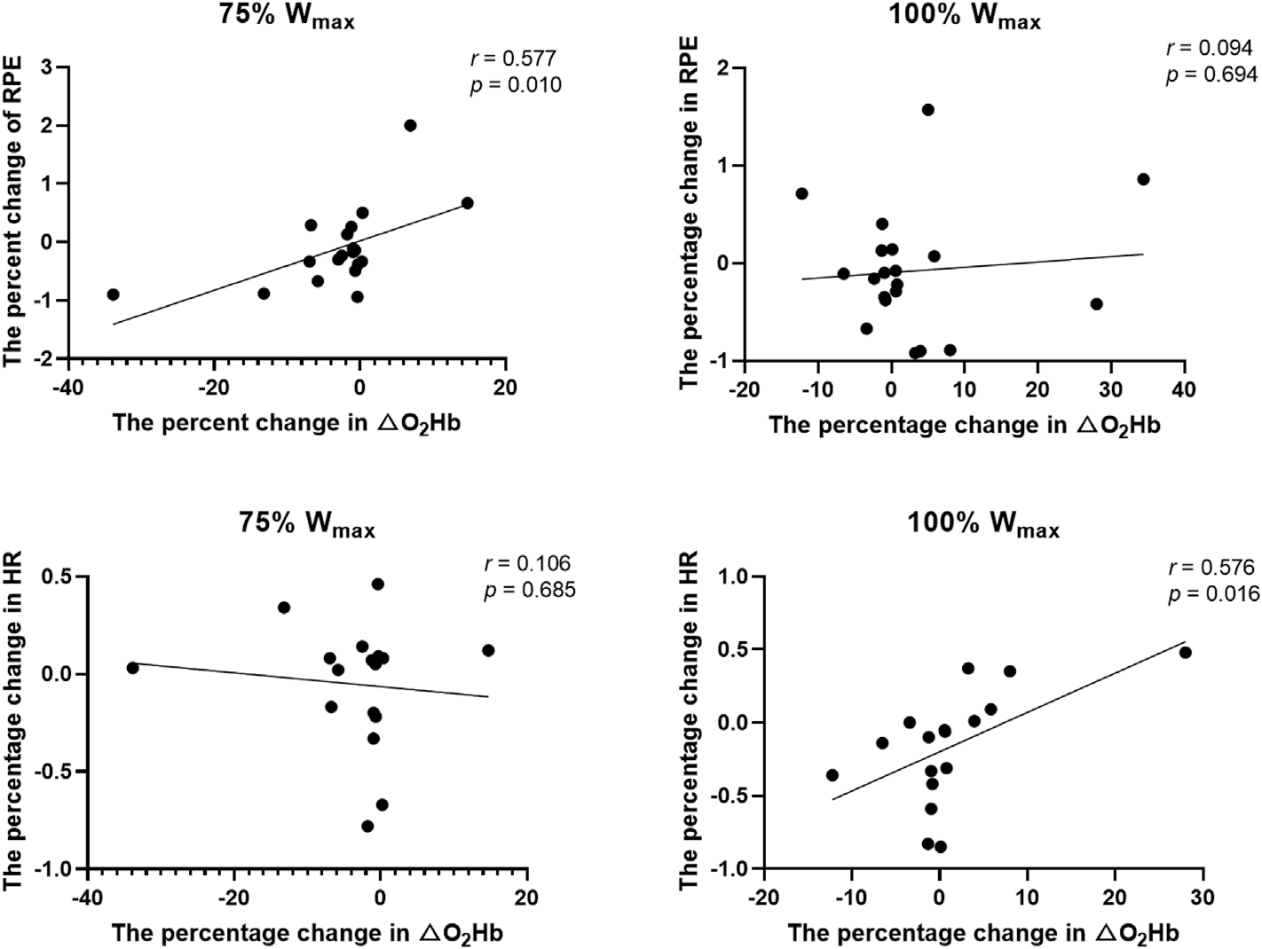
The associations between the H_2_-induced change in the PF and that of the PFC activation. Abbreviations: RPE = ratings of perceived exertion; HR = heart rate; W_max_ = maximum workload.

Based upon the results above, we performed linear regression analysis to examine the associations between the H_2_-induced changes in RPE, HR and △O_2_Hb at 75% W_max_ and 100% W_max_. It was observed that the H_2_-induced change in PF was significantly associated with that in PFC activation, that is, participants with greater percent increase of △O_2_Hb had greater percent reduction in RPE at 75% W_max_ (*r* = 0.577, *p* = 0.010). Similarly, Participants with greater increase of △O_2_Hb had greater percent reduction in HR at 100% W_max_ (*r* = 0.576, *p* = 0.016). No other significant associations were observed (*r* < -0.013, *p* > 0.959) (Figure 3).

## Discussion

This study demonstrated that pre-exercise inhalation of H_2_ gas can alleviate PF and maintain the PFC activation during high-intensity exercise in healthy young adults. More importantly, this study provides first-of-its-kind evidence that the reductions in PF as induced by H_2_ gas was associated with H_2_-induced increase in PFC activation, suggesting a potential “central” pathway that H_2_ may alleviate the PF by altering the activation of brain cortical regions. The findings of this study provide novel insights into the mechanisms underlying the benefits of H_2_ for PF, helping the design of appropriate strategies for fatigue management.

### The anti-fatigue effects of H_2_ gas and its related mechanism

We observed that H_2_ gas attenuated RPE during different phases of task (i.e., different workloads) and could significantly reduce HR under near-maximal workload. These are consistent with the observations in other studies using HRW (Botek et al., 2019; LeBaron et al., 2019; Mikami et al., 2019). Though the inhalation of gas form of H_2_ is currently not a commonly used method, studies have suggested that H_2_ gas can deliver large amounts of H_2_ to the living body quickly through a ventilator circuit or nasal cannula (Kawamura et al., 2020) without risk of explosion even the concentration in the air is below 4% (Ohta, 2011). Furthermore, studies have observed that ingesting H_2_ before exercise can induce anti-fatigue effects and thus benefit endurance capacity (Botek et al., 2019; Mikami et al., 2019). Taken together, we here provide confirmatory but novel evidence that pre-exercise inhalation of H_2_ gas can significantly alleviate PF in healthy participants. Future studies can directly compare the effects of H_2_ gas and HRW, and examine the most appropriate timing of the inhalation of H_2_ (i.e., pre, during or post-exercise) for fatigue attenuation.

It is observed that H_2_ inhalation increased the PFC activation during the high-workload phase, as assessed by △O_2_Hb of fNIRS. The brain is particularly sensitive to ROS (Magistretti and Allaman, 2015) and excessed ROS may damage neurons (Halliwell, 1996) and mitochondrial membranes of the brain, leading to increased proton leakage during mitochondrial energy production (Brookes et al., 1998; Brookes, 2005; Nanayakkara et al., 2019) and reduced efficiency of energy production. H_2_ gas can cross the blood-brain barrier (Hayashida et al., 2012) and selectively scavenge ROS (Ohsawa et al., 2007), helping reduce the damage to neuronal cells. In addition, antioxidant supplements such as resveratrol have been shown to balance redox status and improve brain mitochondrial function (Murphy and Hartley, 2018). Therefore, it may indicate that H_2_ gas could act as a powerful antioxidant to improve brain mitochondrial function and increase energy efficiency by reducing unnecessary oxygen wastage due to proton leakage, resulting in high level of cortical activation during high-intensity exercise. Future studies implementing technique (e.g., respirometry) to assess the bio-neurophysiological characteristics (e.g., mitochondrial function) related to fatigue are needed to explore the underlying pathological mechanism through which the H_2_ can help eliminate the alterations of fatigue on brain functions.

More importantly, we here observed direct association between H_2_-induced improvements in RPE and HR and that in PFC activation. The PFC is the region for the processing of directing, planning and decision making, integrating cognitive and afferent feedback from cardiovascular organs and motor muscles to regulate movement by modulating motor drive (Robertson and Marino, 2016). Specifically, the PFC may have a motivational function that allows continued movement by suppressing those peripheral fatigue signals that want to stop the movement (Miller and Cohen, 2001; Perrey et al., 2016). Therefore, PFC might help delay the onset of fatigue by inhibiting fatigue signals and decreasing subjective effort to increase tolerance to exercise load, leading to lower RPE during exercise. Meanwhile, studies have demonstrated that PFC directs a “neurovisceral integration” system that integrates external environmental stimuli for adaptive regulation of cardiac function, and that increased activation of PFC is associated with enhanced parasympathetic tone (Thayer et al., 2012). Therefore, the observation of decreased HR as induced by H_2_ gas may be due to the enhanced parasympathetic activity via H_2_-induced increase in PFC activation as well.

### The demand to characterize the effects of H_2_ on PF induced by different types of exercise

To note, in several conditions, the inhalation of H_2_ gas did not induce significant improvements on PF (e.g., MVIC), and the associations between the H_2_-induced changes in the activation of PFC and in PF (e.g., changes in △O_2_Hb and RPE at 100% W_max_) were also not significant. This may indicate that the relative contribution of peripheral components to fatigue may increase in these conditions, and more focus on the development/progress of PF in different types of exercises are needed to future characterize the benefits of intaking H_2_ for the alleviation of PF. For example, both high-intensity interval exercise (HIIE) and endurance exercise (EE) can significantly induce PF, but studies have shown that the functional changes (e.g., changes in cardiorespiratory coupling) in response to the PF as induced by these two types of exercise differed (e.g., changes in cardiorespiratory coupling) (Andrade et al., 2020). Specifically, it was observed that the HIIE induced similar changes in muscle mitochondrial function to that induced by EE (Trewin et al., 2018), but may not induce central fatigue (e.g., the central activation ratio of the quadriceps muscle did not change before and after the HIIE) (Jurgelaitiene et al., 2021) which is oftentimes observed after EE (Azevedo et al., 2021). To this point, it is thus highly demanded in future studies to explicitly characterize the effects of different types of exercise protocol on PF and then to more comprehensively characterize the mechanisms through which H_2_ helps alleviate PF resulted from different types of exercise.

### Limitations

Several limitations should be noted in this study. The sample size of participants here was small and only men were recruited; some data were missing or with low quality (e.g., fNIRS). Additionally, only the immediate effects of H_2_ on fatigue and cortical activation were examined. Future studies with a larger sample size and implementing longer term of intervention/exercise are thus highly demanded to examine and confirm the observations in this study and to explore the longer-term effects of repeated inhalation of H_2_ gas. The underlying pathway through which H_2_ gas influences PF and PFC activation needs to be explicitly examined in future studies with more sophisticated biophysiological and biochemical assessments. This will ultimately provide critical knowledge for the optimization of H_2_-based strategies for attenuating PF induced by exercise. Nevertheless, this study provides novel evidence suggesting that the pre-exercise inhalation of H_2_ may help reduce the fatigue during high-intensity exercise via a potential “central” pathway.

## Data Availability

The raw data supporting the conclusions of this article will be made available by the authors, without undue reservation.
